# Prevalence and types of video gaming and gambling activities among adolescent public school students: findings from a cross-sectional study in Italy

**DOI:** 10.1186/s13052-022-01299-2

**Published:** 2022-06-25

**Authors:** Loredana Cena, Matteo Rota, Stefano Calza, Alice Trainini, Sara Zecca, Sofia Bonetti Zappa, Luisa Silvia Nodari, Alberto Stefana

**Affiliations:** 1 Department of Clinical and Experimental Sciences, Section of Neuroscience, Observatory of Perinatal Clinical Psychology, University of Brescia, viale Europa, 11 - 25123 Brescia, Italy; 2 Units of Biostatistics and Biomathematics and Bioinformatics, Department of Molecular and Translational Medicine, University of Brescia, Brescia, Italy; 3 Department of Brain and Behavioural Sciences, University of Pavia, Pavia, Italy

**Keywords:** Video gaming behaviours, Gambling behaviours, Adolescence

## Abstract

Adolescence is characterized by emotional instability and risk-taking behaviours that can lead to, among other things, an increased risk of developing pathological video-gaming and gambling habits. The aim of this Study is to assess the prevalence and type of video gaming and gambling habits in adolescent students attending Italian upper-secondary schools. The cross-sectional study was conducted via an online survey using validated questionnaires. The primary outcome measures were the prevalence of past-year video gaming and gambling activities. The sample consisted of 502 adolescent students from first- and second-grade secondary schools. A total of 40.8% of participants were video gamers, 4.8% were gamblers, 17.8% were both video gamers and gamblers, and the remaining 36.6% were not players. Among participants who reported video gaming activity (*n* = 294), 68.0% were classified as nonproblem gamers, 24.5% as at-risk gamers, and 7.5% as disordered video gamers. Among the participants who reported gambling activity (*n* = 113), 85.8% were not problematic gamblers, 8.9% were at-risk gamblers, and 5.3% were pathological gamblers. Only 0.2% of all subjects met the criteria for both pathological gambling and pathological video game use. The findings indicate that video gaming and gambling are common leisure times among adolescent students. However, a small but significant minority of these adolescents met the criteria for either severe problem gaming or gambling or both.

## Introduction

Adolescence is characterized by emotional instability and risk-taking behaviours that can lead to, among other things, an increased propensity to develop pathological video-gaming and gambling habits.

The eleventh edition of the International Statistical Classification of Diseases and Related Health Problems (ICD-11), which was produced by the World Health Organization, defines gaming disorder as a pattern of recurrent or persistent gaming behaviour manifested by impaired control over gaming, exaggerated priority given to gaming (which takes precedence over daily activities as well as other life interests), and perpetuation or even intensification of gaming despite the occurrence of negative consequences. Furthermore, it defines gambling disorder using the same criteria but referring to gambling instead of gaming behaviours.

Growing empirical evidence suggests that each of these two disorders is positively associated with adolescents’ mental health issues [1, 2], substance use [3, 4], and physical violence [3, 5]. With regard to epidemiologic data, at present, it is estimated that 0.2%-12.3% of adolescents in Europe meet the criteria for internet gaming disorder [6], whereas 0.2%-5.0% meet the criteria for problem gambling [7], with large heterogeneity across nations.

Therefore, it is not surprising that pathological use of video gaming and pathological gambling have become an emerging public health problem [8, 9]. However, to define efficient prevention and intervention plans and to properly allocate resources, a precise estimation of these mental disorders among adolescents remains an urgent requirement. Therefore, the aim of this study was to assess the prevalence and type of video gaming and gambling habits in adolescent students attending Italian upper-secondary schools.

## Methods

### Study design and participants

This is a cross-sectional study with convenient sampling. The participants were recruited from five first-grade secondary public schools (‘middle school’) and five second-grade secondary public schools (‘high school’) located in Brescia Province (Northern Italy). Data collection was carried out from February 2020 until March 2021 through an online survey. A detailed description of the study protocol was published previously [10].

### Measures

The Video-Gaming Scale for Adolescents (VGS-A) and the Gambling Behavior Scale for Adolescents (GBS-A) were used to assess video gaming and gambling behaviours, respectively, that occurred during the last year. They classify the respondents as nonproblem, at-risk, or disordered gamer/gambler. Sociodemographic and educational information were also collected. For a detailed description of the measures, see [11, 12].

### Statistical analysis

Descriptive analyses were performed using R version 4.0.2 (R Foundation for statistical computing, Vienna, Austria).

## Results

A total of 502 adolescent students completed the assessments. The mean age of the participants was 15.9 (SD = 1.93). Most of the participants were female (67.7%), attended high school (79.7%), and had never failed a school year (85.9%). The results indicate that 40.8% of participants were video gamers, 4.8% were gamblers, 17.8% were both video gamers and gamblers, and the remaining 36.6% were not players. More specifically, among participants who reported video gaming activity (*n* = 294), 68.0% were classified as nonproblem gamers, 24.5% as at-risk gamers, and 7.5% as disordered video gamers. On the other hand, among the respondents who reported gambling activity (*n* = 113), 85.8% were not problematic gamblers, 8.9% were at-risk gamblers, and 5.3% were pathological gamblers. Only 0.2% of all subjects met the criteria for both pathological gambling and pathological video game use. The demographic information and prevalence rates of video gaming and gambling activities of adolescent responders to the survey are reported in Table 1.

**Table 1:** Demographics and prevalence of video gaming and gambling activities

	***N* = 502 (100%)**
**Age**, mean (SD)	15.90 (1.93)
**Gender**	
Male	161 (32.1)
Female	340 (67.7)
Other	1 (0.2)
**Area of residence**	
Downtown	84 (16.7)
Suburbs	139 (27.7)
Countryside	112 (22.3)
Mountain	79 (15.7)
Lake	88 (17.5)
**School class**	
3rd lower secondary school	107 (21.3)
1st upper secondary school	43 (8.6)
2nd upper secondary school	74 (14.7)
3rd upper secondary school	79 (15.7)
4th upper secondary school	105 (20.9)
5th upper secondary school	94 (18.7)
**History of at least one school failure**, Yes	71 (14.1)
**Gambling activity**	
Non-problematic gambler	97 (19.3)
At-risk gambler	10 (2.0)
Disordered gambler	6 (1.2)
Non-gambler	
**Video gaming use**	
Non-problematic gamer	200 (39.8)
At-risk gamer	72 (14.3)
Disordered gamer	22 (4.4)
Non-gamer	208 (41.4)
**Interrelations between gaming and gambling activities**	
Not a gamer nor gambler	
Non-problematic gamer and gambler	54 (10.8)
Not a gamer but at-risk gambler	1 (0.2)
Not a gamer but disordered gambler	1 (0.2)
Not a gambler but at-risk gamer	50 (10.0)
Not a gambler but disordered gamer	18 (2.6)
At-risk gamer and gambler	2 (0.4)
Disordered gamer and gambler	2 (0.4)
Non-problematic gamer and non-gambler	142 (28.2)
Non-problematic gambler and non-gamer	22 (4.4)
Non-problematic gamer and at-risk gambler	4 (0.8)
Non-problematic gambler and at-risk gamer	18 (3.6)
Non-problematic gamer and disordered gambler	3 (0.6)
At-risk gamer and disordered gambler	2 (0.4)
At-risk gambler and disordered gamer	4 (0.8)

## Discussion and conclusion

Our findings indicate that video gaming and, to a lesser extent, gambling are common leisure activities among adolescents, and were reported by approximately half and one-fourth of the students surveyed, respectively. However, a small but significant minority (5.0%) of these adolescents met the criteria for either severe problem gaming, severe problem gambling or both. The evidence presented here is consistent with international literature [7, 8] and will hopefully encourage more research into youth video gaming and gambling to better elucidate the determinants of these phenomena.

The major limitations of this study are the cross-sectional design, the use of self-report tools, and a limited sample size that did not allow the use of logistic regression analysis to determine the specific odds for various vulnerability and protective factors.

Future studies should expand the sample size and include adolescents who do not attend school and represent different parts of the country. Assessing the prevalence of problematic video gaming use and gambling in adolescents would help to increase awareness about these emergent public health issues and take specific measures for preventing, identifying, managing, and treating these disorders.

## Data Availability

The datasets used and analyzed during the current study are available from the corresponding author on reasonable request.

## Abbreviations

ICD-11: International Statistical Classification of Diseases and Related Health Problems
VGS-A: Video-Gaming Scale for Adolescents
GBS-A: Gambling Behavior Scale for Adolescents
SD: Standard Deviation
